# Modulation of Low-Voltage-Activated Inward Current Permeable to Sodium and Calcium by DARPP-32 Drives Spontaneous Firing of Insect Octopaminergic Neurosecretory Cells

**DOI:** 10.3389/fnsys.2017.00031

**Published:** 2017-05-19

**Authors:** Bruno Lapied, Antoine Defaix, Maria Stankiewicz, Eléonore Moreau, Valérie Raymond

**Affiliations:** ^1^Laboratoire SiFCIR UPRES EA 2647/USC INRA 1330, Université Bretagne Loire, University of Angers, UFR SciencesAngers, France; ^2^Faculty of Biology and Environment Protection, N. Copernicus UniversityTorun, Poland

**Keywords:** DUM neurons, pacemaker activity, DARPP-32, octopamine, low-voltage-activated current

## Abstract

Identification of the different intracellular pathways that control phosphorylation/dephosphorylation process of ionic channels represents an exciting alternative approach for studying the ionic mechanisms underlying neuronal pacemaker activity. In the central nervous system of the cockroach *Periplaneta americana*, octopaminergic neurons, called dorsal unpaired median (DUM; DUM neurons), generate spontaneous repetitive action potentials. Short-term cultured adult DUM neurons isolated from the terminal abdominal ganglion (TAG) of the nerve cord were used to study the regulation of a tetrodotoxin-sensitive low-voltage-activated (LVA) channel permeable to sodium and calcium (Na/Ca), under whole cell voltage- and current-clamp conditions. A bell-shaped curve illustrating the regulation of the amplitude of the maintained current vs. [ATP]i was observed. This suggested the existence of phosphorylation mechanisms. The protein kinase A (PKA) inhibitor, H89 and elevating [cyclic adenosine 3′, 5′ monophosphate, cAMP]i, increased and decreased the current amplitude, respectively. This indicated a regulation of the current via a cAMP/PKA cascade. Furthermore, intracellular application of PP2B inhibitors, cyclosporine A, FK506 and PP1/2A inhibitor, okadaic acid decreased the current amplitude. From these results and because octopamine (OA) regulates DUM neuron electrical activity via an elevation of [cAMP]i, we wanted to know if, like in vertebrate dopaminergic neurons, OA receptor (OAR) stimulation could indirectly affect the current via PKA-mediated phosphorylation of Dopamine- and cAMP-regulated Phosphoprotein-32 (DARPP-32) known to inhibit PP1/2A. Experiments were performed using intracellular application of phospho-DARPP-32 and non-phospho-DARPP-32. Phospho-DARPP-32 strongly reduced the current amplitude whereas non-phospho-DARPP-32 did not affect the current. All together, these results confirm that DARPP-32-mediated inhibition of PP1/2A regulates the maintained sodium/calcium current, which contributes to the development of the pre-depolarizing phase of the DUM neuron pacemaker activity.

## Introduction

Pacemaker neurons are well characterized by their intrinsic ability to generate spontaneous beating or bursting overshooting action potentials. Generation of spontaneous rhythmic activity involved special class of ionic currents occurring during the interval between spikes (Bean, 2007). Among voltage-gated ion currents underlying the neuronal pacemaker activity, the hyperpolarization-activated cyclic-nucleotide-gated cation non-selective channels, HCN1–4, (Robinson and Siegelbaum, 2003; Santoro and Baram, 2003; He et al., 2014; Cao et al., 2016), activated at subthreshold potentials play crucial roles to establish pacemaker potential.

In addition, T-type channels are known to also shape the firing properties. This low-voltage-activated (LVA) transient calcium current is able to activate from small depolarizations near the resting membrane potential and can generate spontaneous electrical activity (Kostyuk, 1999; Perez-Reyes, 2003; Cueni et al., 2009; Cain and Snutch, 2013; Cheong and Shin, 2013; Lambert et al., 2014; Turner and Zamponi, 2014). Three genes encoding the T-type channel alpha subunit have been identified (Cav 3.1, Cav3.2 and Cav3.3; Perez-Reyes, 2003). The calcium currents generated by Cav3.3 subunit displays slower kinetics that differs from the kinetics observed for Cav3.1 and Cav3.2 (Lacinová et al., 2000). This confirms the existence of a native neuronal sustained calcium current, also considered as member of the LVA calcium channel group. This current, previously described in insect octopaminergic neurons, named the dorsal unpaired median (DUM) neurons is activated with small depolarizations, controls the frequency and pattern of DUM neuron spikes (Avery and Johnston, 1996; Grolleau and Lapied, 1996, 2000; Kostyuk, 1999; Wicher et al., 2001; Heidel and Pflüger, 2006). In addition, another depolarization-activated inward current identified as low-threshold persistent sodium currents also contribute to neuronal excitability in vertebrate as well as in insect neuronal preparations (Lapied et al., 1990; Crill, 1996; Grolleau and Lapied, 2000; Jackson et al., 2004; Yamada-Hanff and Bean, 2013; Deng and Klyachko, 2016; Paul et al., 2016). Although the molecular nature of the channels carrying persistent sodium current seems unclear, the persistent sodium current could be carried by fraction of sodium channels that fails to inactivate (Taverna et al., 1999) or the persistent sodium current could arise from the incomplete inactivation of the fast sodium current (Crill, 1996; Taddese and Bean, 2002). Finally and besides this myriad of LVA currents, another less known LVA maintained inward current permeable to both sodium and calcium (Na/Ca) has been characterized in DUM neurons (Defaix and Lapied, 2005). This mixed conductance is active and does not inactivate at sub-threshold voltages and plays a critical role in setting DUM neuron excitability. Because intracellular signaling pathways are essential in regulating ion channel functions, an essential missing functional consideration emerges linking intracellular signaling mechanisms and electrical signaling where the opening and closing of ion channels control the neuron’s firing rate. In insects, one of the most prominent biogenic amine in the nervous system is octopamine (OA), known to act as a neurotransmitter, neuromodulator and neurohormone (Evans and Maqueira, 2005; Roeder, 2005). Although it is well known that OA is released from a small number of identified neurosecretory cells, the DUM neurons, clustered along the dorsal midline of all ganglia (except from brain) of the ventral nerve cord (Bräunig and Pflüger, 2001), OA is also highly functionally significant, which may strongly influence electrical behaviors and signals produced by DUM neurons (Achenbach et al., 1997; Wicher et al., 2001). However, so far, the OA-induced activation of the complex intracellular signaling pathways involved in such regulation is still elusive. Understanding how intracellular biochemical networks and electrical activity are integrated is an essential ongoing question to go deeper in the DUM neuron physiological functions. Using whole cell patch-clamp technique and immunocytochemistry, we have studied the regulatory role of the biogenic amine, OA on the LVA maintained inward current permeable to both Na/Ca involved in the generation of the pacemaker potential. These findings lead us to propose a novel control of the neuronal pacemaker mechanism.

## Materials and Methods

### Preparation

All experiments were performed on DUM neurons cell bodies isolated from the dorsal midline of the terminal abdominal ganglion (TAG) of the ventral nerve cord of adult male cockroach *Periplaneta americana*, reared under standard conditions (29°C, photocycle of 12 h light/12 h dark). Insects were anesthetized by cold treatment. Animal care and handling procedures were in accordance with French institutional and national health guidelines. Cockroaches were pinned dorsal side up on a dissection dish. The dorsal cuticules were removed to allow access to the ventral nerve cord. The TAG were then carefully dissected under a binocular microscope and placed in normal cockroach saline containing (in mM): NaCl 200, KCl 3.1, CaCl_2_ 5, MgCl_2_ 4, sucrose 50, HEPES 10; pH was adjusted to 7.4 with NaOH.

### Cell Isolation

Isolation of DUM neuron cell bodies was performed under sterile conditions using enzymatic treatment and mechanical dissociation of the median part of the TAG, as previously described (Lapied et al., 1989). DUM neurons were maintained at 29°C for 24 h before electrophysiological experiments were carried out. The DUM neuron cell bodies used in the present study were chosen as indicated previously (Lapied et al., 1989).

### Whole Cell Recording and Data Analysis

We used the patch-clamp technique in the whole cell configuration to record spontaneous electrical activity and membrane currents. Patch pipettes were pulled from borosilicate glass capillary tubes (GC 150 T-10, Harvard Apparatus, Edenbridge, UK) with a PP-83 electrode puller (Narishige, Tokyo, Japan). Pipettes had resistances ranging from 0.7 MΩ to 1.3 MΩ when filled with internal solution (see composition below). The liquid junction potential between the pipette and the superfusing solution was always corrected before formation of a seal ≥2 GΩ. Signals were recorded with an Axopatch 200A amplifier (Axon Instruments, Foster City, CA, USA). Electrical commands were generated by a programmable stimulator (SMP 310, Biologic, Claix, France) or an IBM computer (Pentium 100) with software control pClamp 8.0.2 connected to a 16-bit analog-to-digital converter (Digidata 1322A, Axon Instruments). Although leak and capacitive currents were compensated electronically at the beginning of each experiment, subtraction of residual capacitance and leakage currents was performed with an on-line P/4 protocol provided by pClamp. In this procedure, currents elicited by four subpulses from the holding potential with an amplitude one-fourth of the main pulse were added together to compute capacitance and leak-subtracted currents. Serie resistance value was obtained by the amplifier for each experiment from the patch-clamp amplifier settings after compensation and varied between 2 MΩ and 3 MΩ. Cells were clamped at a holding potential of –90 mV and 100 ms test pulses (except when otherwise stated) were applied from the holding potential at a frequency of 0.14 Hz. For current-clamp experiments, action potentials were displayed on a digital oscilloscope (310, Nicollet Instruments, Madison, WI, USA) and stored on a DAT (DTR 1202, Biologic) or on the hard disk of the computer for subsequent off-line analysis.

### Immunocytochemistry

For light microscope immunocytochemistry, isolated DUM neuron cell bodies were fixed for 1 h in 4% paraformaldehyde containing 5% (wt/vol) sucrose in phosphate-buffered saline (PBS). To block non specific binding of the primary antibody, isolated DUM neuron cell bodies were incubated with 4% bovine serum albumin (BSA) in PBS containing 0.2% Triton X-100 for 1 h. Primary antiserum (rabbit anti-cyclic AMP, Chemicon International, Temecula, CA, USA) diluted 1/800 in 0.2% Triton X-100 in PBS was applied for 12 h at 4°C. After repeated washing in PBS, the secondary antibody (FITC-labeled goat anti-rabbit IgG, Chemicon International) diluted 1/300 in PBS containing 1% BSA and 0.2% Triton X-100 was applied for 3 h at 20°C in the dark. Isolated DUM neuron cell bodies were then washed in 4% BSA in PBS and mounted on glass slides in glycerol-PBS. Control experiments were performed by omitting primary antibodies. Preparations were viewed and photographed through a Zeiss Axioscope microscope (Germany) with an epifluorescence system. Images were digitized with Axovision software.

### Solutions

The solutions used to record whole cell inward current were designed to eliminate interference from potassium currents by the combination of external 4-aminopyridine (4-AP) and tetraethylamonium-chloride (TEA-Cl) and by isotonically substituting potassium with cesium in the patch electrode. Inward calcium currents were abolished by adding external 0.5 mM CdCl_2_. The extracellular solution superfusing the cell contained (in mM): NaCl 100, TEA-Cl 100, KCl 3.1, CaCl_2_ 2, MgCl_2_ 7, CdCl_2_ 0.5, 4-AP 3, HEPES 10; pH was adjusted to 7.4 with TEA-OH. For all voltage-clamp experiments, the patch pipette solution contained (in mM): CsCl 90, CsF 80, NaCl 15, MgCl_2_ 1, EGTA 5, HEPES 10, ATP-Mg 1 (except when otherwise stated); pH was adjusted to 7.4 with CsOH. For the determination of the physiological role of the current, the bath solution contained (in mM): NaCl 100, TEA-Cl 100, KCl 3.1, CaCl_2_ 5, MgCl_2_ 4, NiCl_2_ 0.1, 4-AP 3, HEPES 10. In this experiment, patch electrodes were filled with an internal solution containing (in mM): CsCl 90, CsF 80, NaCl 15, MgCl_2_ 1, EGTA 10, CaCl_2_ 0.5, HEPES 20, ATP-Mg 1; pH was adjusted to 7.4 with CsOH. For current-clamp recordings, the patch pipette solution contained (in mM): K aspartate 160, KF 10, NaCl 10, MgCl_2_ 1, CaCl_2_ 0.5, EGTA 10, HEPES 10; pH was adjusted to 7.4 with KOH. The bathing solution was the normal cockroach saline. Phosphorylated and non-phosphorylated recombinant Dopamine- and cAMP-regulated Phosphoprotein-32 (DARPP-32) were a generous gift of S. N. Schiffmann. Recombinant rat DARPP-32 was expressed in *Escherichia coli* using pEt-3A vector, purified and prepared as previously described (Neyroz et al., 1993; Schiffmann et al., 1998). The two compounds were used at 0.3 mg/mL. All chemical products were purchased from Sigma-Aldrich (L’isle d’Abeau Chesnes, France) except NaCl, KCl, sucrose and MgCl_2_ (Merck Eurolab SA, Fontenay sous bois, France), DL OA hydrochloride (Fluka, L’isle d’Abeau Chesnes, France). Experiments were carried out at room temperature (20°C). Data, when quantified, were expressed as mean ± SEM. Differences between means were tested for statistical significance by Student’s *t*-test.

## Results

### The LVA Maintained Na/Ca Inward Current Is Regulated by Intracellular ATP Concentration

**Figure 1:**
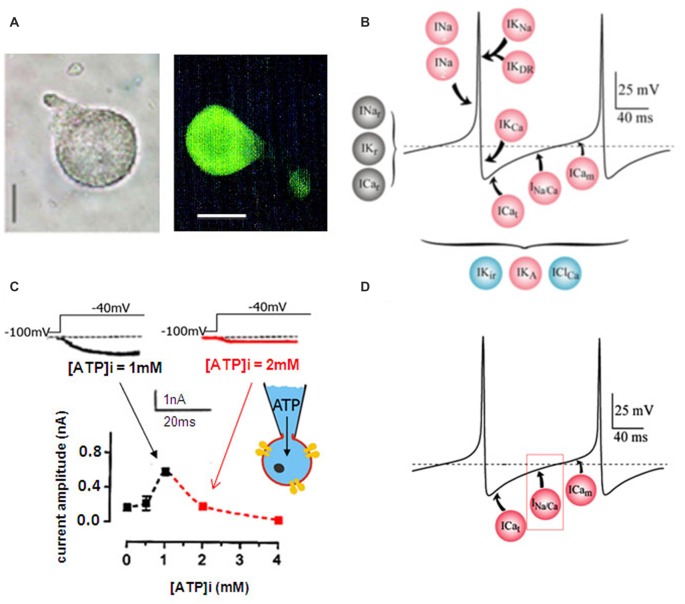
**Dorsal unpaired median (DUM) neuron cell body isolated form the terminal abdominal ganglion (TAG) of the cockroach** ***Periplaneta americana***. **(A)** Light micrographs of an isolated DUM neuron cell body maintained in short-term culture and treated with a polyclonal antibody directed against octopamine (OA). Similar results were obtained in 12 different cells. Scale bars, 40 μm. **(B)** Model representing the ionic currents involved in the generation of the different phases of the beating pacemaker activity. Pink dots, represent the depolarization-activated currents, blue dots, the hyperpolarization-activated currents and the black ones illustrate the resting currents. **(C)** Effect of intracellular ATP concentration on the low-voltage-activated (LVA) maintained sodium and calcium (Na/Ca) inward current. Typical examples of inward current traces recorded in the presence of 1 mM ATP (black trace) and 2 mM ATP (red trace) added in the pipette solution and elicited by a 30-ms depolarizing pulse to −40 mV from a holding potential of −100 mV. The graph illustrated the effects of low and high intracellular ATP concentration on the LVA maintained Na/Ca inward current amplitude recorded at test pulse of −40 mV from a holding potential of −100 mV. **(D)** Hypothetic model illustrating the physiological implication of the LVA maintained Na/Ca inward current in the generation of the pre-depolarizing phase of the DUM neuron pacemaker activity. ICa_t_ and ICa_m_ represent the LVA transient and maintained calcium currents, respectively.

All experiments were performed on isolated adult DUM neuron cell body exhibiting OA-like immunoreactivity (Figure 1A) and known to generate endogenous pacemaker activity even in the absence of rhythmic somatic input, which is dependent on multiple different voltage-gated currents and background currents (Figure 1B; Grolleau and Lapied, 2000; Wicher et al., 2001). This study was mainly focused on the regulation of a novel LVA maintained inward current permeable to Na/Ca and involved in the generation of the pre-depolarizing phase of the pacemaker activity (Figure 1D; Defaix and Lapied, 2005). We previously determined experimental conditions allowing full activation of the maintained Na/Ca current (Defaix and Lapied, 2005). As illustrated in the Figure 1C, we used different intracellular solutions with increasing ATP concentration from 0 mM to 4 mM. The amplitude of the maintained current was maximum for 1 mM [ATP]_i_ and decreased for lower and higher [ATP]_i_ (Figure 1C). Based on the [ATP]_i_-induced bell-shaped modulation of the Na/Ca current amplitude, the intracellular ATP concentration of 1 mM was chosen as control conditions for all this study.

### Phosphorylation/Dephosphorylation Process Regulates the LVA Maintained Inward Na/Ca Current in DUM Neurons

**Figure 2:**
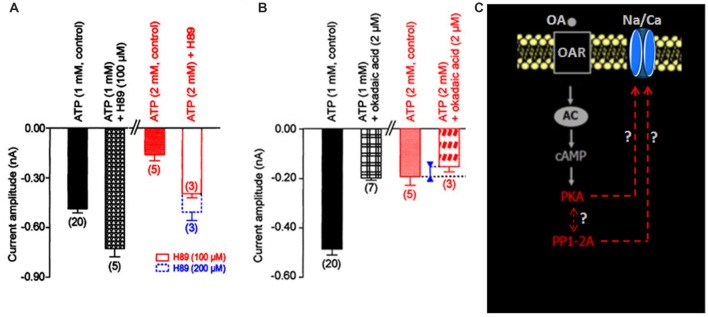
**Effects of internal application of H89 and okadaic acid on the LVA maintained Na/Ca inward current amplitude. (A,B)** Histograms summarizing the mean current amplitude recorded at −40 mV from a holding potential of −100 mV under different experimental conditions indicated above each bar. Data are means ± SEM. Values in parentheses indicate number of experiments in each condition. **(C)** Hypothetic model illustrating the participation of the molecular events identified as protein kinase A (PKA) and protein phosphatase (PP1/2A; red characters) in the regulation of the LVA maintained Na/Ca inward current amplitude.

The participation of the cyclic adenosine 3′, 5′ monophosphate (cAMP-dependent) protein kinase A (PKA) in the regulation of the Na/Ca current was suggested by the significant sensitivity of the current to intracellular ATP concentration (Figure 1C). In control conditions (i.e., [ATP]_i_: 1 mM), the PKA inhibitor, H89 (100 μM) increased the current amplitude from –0.49 ± 0.03 nA (control, *n* = 20) to –0.73 ± 0.05 nA (*n* = 5; *p* < 0.05; Figure 2A). When the patch pipette solution contained 2 mM [ATP]_i_, the decreased current amplitude (to –0.13 ± 0.01 nA (*n* = 4; *p* < 0.01) observed was dose-dependent reversed with 100 μM and 200 μM H89_i_ (current amplitudes were −0.37 ± 0.03 nA (*n* = 3) and –0.48 ± 0.05 nA (*n* = 3)), respectively (Figure 2A). These results illustrating that H89 was able to abolish the inhibitory effect of PKA suggesting a negative regulatory action of PKA activation on the Na/Ca inward current.

To check whether a protein phosphatase was involved in the reversal of the phosphorylated Na/Ca channel, intracellular application of the potent protein phosphatase inhibitor okadaic acid, known to inhibit protein phosphatases PP1/2A (Herzig and Neumann, 2000) was tested on the inward current. Experiments performed with the patch pipette solution containing 1 mM [ATP]_i_, revealed that okadaic acid (2 μM) decreased the current amplitude from –0.49 ± 0.03 nA (*n* = 20) to –0.18 ± 0.04 nA (*n* = 3; *p* < 0.01; Figure 2B). In the presence of 2 mM [ATP]_i_, the current amplitude only slightly decreased from –0.19 ± 0.04 nA (*n* = 5) to –0.15 ± 0.02 nA (*n* = 3). These results indicated that the phosphatase PP1/2A was also involved in the regulation of the Na/Ca current and that PP1/2A was obviously inhibited when PKA was activated (i.e., with [ATP]_i_ 2 mM, see Figure 2A). In other words, we revealed that the dephosphorylation mechanism via an okadaic acid-sensitive phosphatase could have important functional consequences on such DUM neuron Na/Ca channels particularly when PKA was activated. According to the hypothetical scheme shown in Figure 2C, we proposed that the Na/Ca channel existed either in the phosphorylated or dephophosphorylated state. Intracellular ATP concentration regulated the Na/Ca current amplitude by activating PKA, which phosphorylated the molecule and maintained Na/Ca channels in nonfunctional form. Phosphorylation was opposed by a dephosphorylation process, which rendered the channel functional.

### The LVA Maintained Inward Na/Ca Current Is Modulated by OA via a cAMP/PKA Cascade

**Figure 3:**
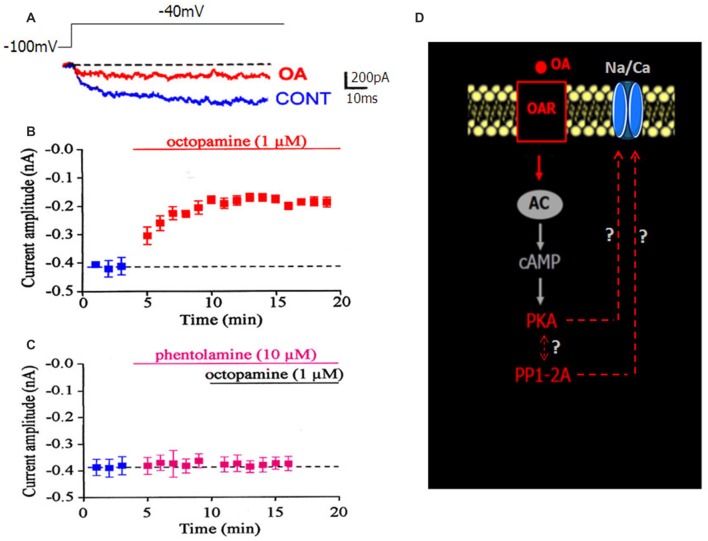
**Effect of OA on the LVA maintained Na/Ca inward current. (A)** Superimposed current traces generated by depolarizing pulse to −40 mV from a holding potential of −100 mV and recorded in control condition (blue current trace) and in the presence of 1 μM OA (red current trace). **(B)** Time course of changed maximum current amplitude induced by bath application of 1 μM OA. **(C)** Pretreatment of DUM neuron cell body by the OA receptor (OAR) antagonist, phentolamine (10 μM), completely blocked the OA-induced decrease current amplitude. In both cases, data are means ± SEM. **(D)** Hypothetic scheme illustrating the involvement of OAR activated by OA (red characters) in the modulation of the LVA maintained Na/Ca inward current amplitude.

**Figure 4:**
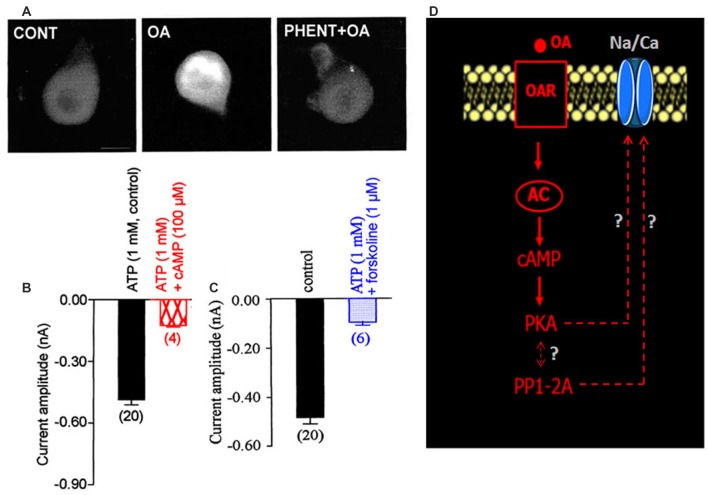
**Involvement of adenylyl cyclase (AC) activity in the OA-induced modulation of the LVA maintained Na/Ca inward current. (A)** Cyclic adenosine 3′,5′ monophosphate (cAMP) immunoreactivity of isolated DUM neuron cell bodies. Comparative change in immunofluorescence observed in control and after application of 1 μM OA. After pretreatment of DUM neuron cell body with 10 μM phentolamine, the OAR antagonist, cAMP immunostaining induced by OA was completely abolished (*n* = 8, in each experimental conditions). **(B,C)** Comparative histograms illustrating the mean current amplitude recorded at −40 mV from a holding potential of −100 mV under different experimental conditions indicated above each bar. Data are means ± SEM. Values in parentheses indicate number of experiments in each condition. **(D)** Hypothetic model indicating the participation of the increased cAMP concentration via AC activated by the action of OA on OAR, which modulates the LVA maintained Na/Ca inward current.

**Figure 5:**
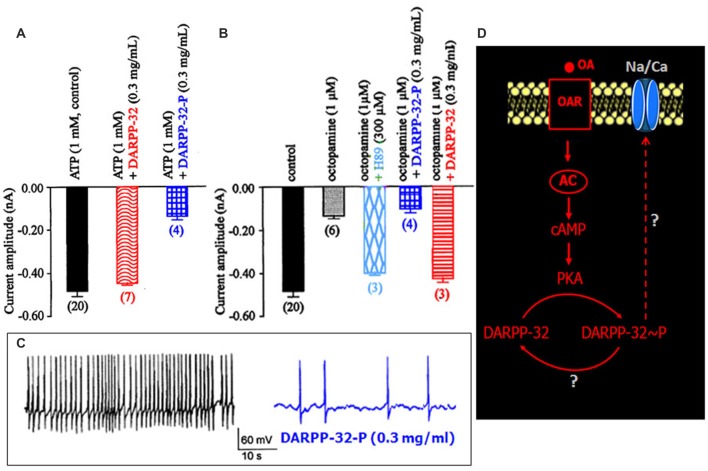
**Effects of dephosphorylated and phosphorylated Dopamine- and cAMP-regulated Phosphoprotein-32 (DARPP-32-P) on the LVA maintained Na/Ca inward current. (A,B)** Comparative histograms summarizing the mean current amplitude recorded at −40 mV from a holding potential of −100 mV under different experimental conditions indicated above each bar. Data are means ± SEM. Values in parentheses indicate number of experiments in each condition. **(C)** Effect of DARPP-32-P on spontaneously active DUM neuron. DARPP-32-P (0.3 mg/mL) decreases action potential discharge frequency (similar results were obtained in six different cells). **(D)** Hypothetic model illustrating the implication of DARPP-32 and DARPP-32-P in the modulation of the LVA maintained Na/Ca inward current produced by OA through cAMP/PKA cascade.

DUM neurons are insect neurosecretory cells whose pacemaker electrical activity is modulated by OA (Achenbach et al., 1997; Wicher et al., 2001). According to these previous findings, we performed additional experiments to study the potential effect of OA on the LVA maintained Na/Ca current, known to play a crucial role in the generation of the DUM neuron pacemaker activity (Figure 1D). When OA (1 μM) was bath applied onto isolated DUM neuron cell body, an important decrease of the current amplitude was observed (from –0.49 ± 0.03 nA in control, (*n* = 20) to −0.17 ± 0.01 nA (*n* = 6; *p* < 0.01), measured at *t* = 14 min, Figures 3A,B, 5B), which was very close to the current amplitude recorded under experimental conditions where PKA was activated (see Figure 2A). To confirm whether OA receptors (OARs) were involved in the OA-induced regulatory effect of the Na/Ca current, we applied phentolamine, a well-known OAR antagonist. As illustrated in Figure 3C, the effect of OA was completely abolished by phentolamine (10 μM). As hypothesized in the summarizing scheme shown in Figure 3D, the effects of OA are thought to be mainly mediated by interaction with G-protein coupled receptors, which trigger, for instance, activation of the cAMP/PKA cascade (Evans and Maqueira, 2005; Farooqui, 2007). Based on our findings, to study if the action of OA was coupled to increases in intracellular levels of the second messenger cAMP, antibodies raised against cAMP (De Vente et al., 1993) were used. As shown in Figure 4A, pretreatment with phentolamine (10 μM) abolished the intensity of fluorescent cAMP immunostaining produced by OA. These results provided evidence that the action of OA on the Na/Ca current involved the rise in internal cAMP level. To more deeply explore this hypothesis, DUM neurons were dialyzed using an internal solution containing 100 μM cAMP. When intracellular cAMP (100 μM) was introduced into cell body by diffusion through the patch pipette, the maximum current amplitude was decreased from –0.49 ± 0.03 nA (control, *n* = 20) to −0.161 ± 0.007 nA (100 μM cAMP added in the patch pipette, *n* = 4; *p* < 0.01; Figure 4B). The effects of regulating PKA phosphorylation were monitored by comparing the amplitude of the Na/Ca current before (standard conditions) and after external application of forskoline. Figure 4C shows that application of forskoline (1 μM), which directly activates adenylyl cyclase (AC), produced a decrease in current amplitude from –0.49 ± 0.03 nA (control, *n* = 20) to −0.13 ± 0.01 nA (*n* = 6; *p* < 0.01). As previously indicated, OA decreased the current amplitude, which was reversed by 300 M H89 (Figure 5B). Because this effect was mimicked by a relative high cAMP internal concentration (100 μM) and by forskoline and blocked by H89 (Figure 2A), we assumed it occurred through cAMP/PKA cascade via the activation of AC (Figure 4D).

### Modulation of the LVA Maintained Na/Ca Current in DUM Neurons by the Phosphoprotein DARPP-32

**Figure 6:**
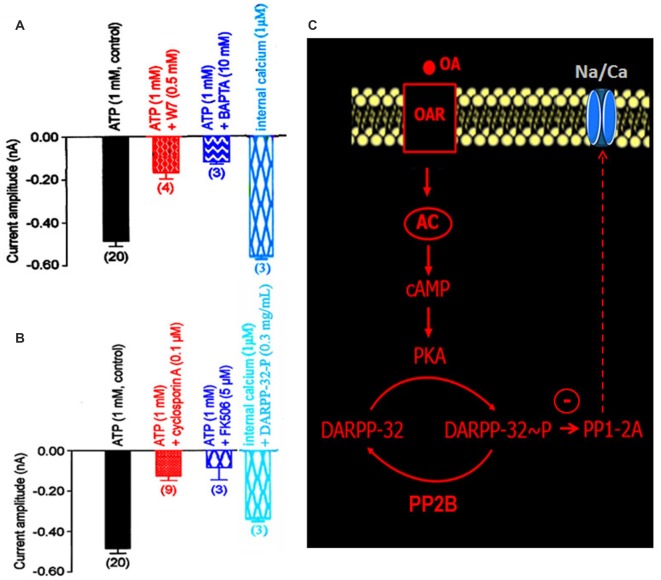
**Implication of the calcium/calmodulin-sensitive protein phosphatase PP2B in the DARPP-32/DARPP-32-P-induced modulation of the LVA maintained Na/Ca inward current. (A,B)** Comparative histograms illustrating the current amplitude recorded at −40 mV from a holding potential of −100 mV under different experimental conditions indicated above each bar. Data are means ± SEM. Values in parentheses indicate number of experiments in each condition. **(C)** Summarizing hypothetical model suggesting that PP2B activation dephosphorylated DARPP-32-P. By contrast, the phosphorylation of DARPP-32 via the activation of PKA produced an inhibition of the okadaic acid-sensitive protein phosphatase PP1/2A, which threreby modulated the LVA maintained Na/Ca inward current.

DARPP-32 is an important mediator of biogenic amines in vertebrate neurons. It is now assumed that DARPP-32 plays a crucial role as an integrator to diverse neurotransmission inputs in vertebrates (Svenningsson et al., 2004). Based on our results, and because the phosphorylation states of DARPP-32 are affected by a number of neurotransmitters such as dopamine and serotonin, it is tempting to hypothesize that such phosphoprotein complexes might be involved in the OA-induced modulation of the Na/Ca current in DUM neurons. The phosphorylated and non-phosphorylated forms of DARPP-32 (0.3 mg/mL) were then tested on the amplitude of the Na/Ca current (Figures 5A,B). Application of the phosphorylated form of DARPP-32 (DARPP-32-P) decreased the Na/Ca current amplitude, from –0.49 ± 0.03 nA (control, *n* = 20) to –0.16 ± 0.02 nA (*n* = 4; *p* < 0.01) whereas application of the non-phosphorylated form of DARPP-32 had no significant effect on the current amplitude (−0.45 ± 0.01 nA, *n* = 7; *p* > 0.05; Figure 5A). OA, which is expected to act via the phosphoprotein DARPP-32, was tested in the presence of the non-phosphorylated form of DARPP-32 (0.3 mg/mL). As indicated above, OA alone strongly decreased the Na/Ca current amplitude (Figure 5B). By contrast, application of OA, in the presence of excess DARPP-32 had no significant effect on the current amplitude (−0.43 ± 0.02 nA (*n* = 3; *p* > 0.05) vs. –0.49 ± 0.03 nA (*n* = 20). This effect was very similar to that of observed with H89 (Figure 5B). It should be noted that when DUM neuron cell body was pretreated with DARP-32-P, which already reduced current amplitude (Figure 5A), OA (1 μM) did not produce any additional effect on the Na/Ca current (Figure 5B). In addition, the physiological role of DARPP-32-P was directly assessed on spontaneously active DUM neurons. As expected, from previous data reporting the involvement of the Na/Ca current in the pre-depolarizing phase of the pacemaker activity (Defaix and Lapied, 2005), the frequency of firing was strongly decreased in the presence of DARPP-32-P (0.3 mg/mL; from 1.4 ± 0.4 Hz to 0.11 ± 0.05 Hz, *n* = 6; Figure 5C). These results indicated that upon activation of OARs DARPP-32 was phosphorylated by PKA, via the cAMP/PKA cascade (Figure 5D). In this case and according to the literature, phosphorylation turned DARPP-32 into a potential potent inhibitor of PP1/2A. This was confirmed by experiments performed with the PP1/2A inhibitor, okadaic acid. Finally, another aspect of the DARPP-32P/P1–2A cascade was that DARPP-32-P was dephosphorylated by the calcium/calmodulin-dependent protein phosphatase PP2B. To check whether PP2B was also involved in the modulatory effect of the Na/Ca current, additional set of experiments were performed with intracellular application of BAPTA, a fast efficient calcium chelator and W7, the calmodulin inhibitor. As illustrated in Figure 6A, both W7 (0.5 mM) and BAPTA (10 mM) produced a strong decrease of the current amplitude (from –0.49 ± 0.03 nA (*n* = 20) to –0.19 ± 0.03 nA (*n* = 4) and to –0.11 ± 0.01 nA (*n* = 3), respectively; *p* < 0.01). By contrast, high intracellular calcium concentration (1 μM) added in the pipette solution slightly increased current amplitude. To substantiate the involvement of PP2B, experiments were also carried out with cyclosporin A (0.1 μM) and FK506 (5 μM), two well-know blockers of PP2B. Once again, both compounds reduced the Na/Ca current amplitude from –0.49 ± 0.03 nA (*n* = 20) to −0.17 ± 0.02 nA (*n* = 9) and to –0.09 ± 0.06 nA (*n* = 3), respectively (Figure 6B; *p* < 0.01). It should be mentioned that the amplitude of the Na/Ca current increased following elevation of intracellular calcium concentration (1 μM). This effect was not observed in the presence of FK506 (5 μM; not shown) and was only reduced in the presence of excess DARPP-32-P (Figure 6B). Taken all together these results, we proposed the final hypothetical scheme, which summarized the different molecular events involved in the OA-induced modulation of the Na/Ca inward current occurring through the phosphoprotein DARPP-32 (Figure 6C).

## Discussion

Octopaminergic DUM neurons project their axons both centrally, innervating neuropiles of different ganglia but also peripherally to innervate skeletal and visceral muscles and some sense organs. It is also well established that DUM neurons are an important component of different motor networks (Burrows and Pflüger, 1995; Baudoux et al., 1998; Mentel et al., 2008; Vierk et al., 2009). Although DUM neurons may be activated by sensory stimuli (e.g., Baudoux and Burrows, 1998; Field et al., 2008; Pflüger et al., 2011; Rand et al., 2012), they are defined by the absence of common somatic synaptic inputs from presynaptic neurons and by their uncommon intrinsic property allowing adequate beating pacemaker activity (Grolleau and Lapied, 2000; Wicher et al., 2001; Defaix and Lapied, 2005; Heidel and Pflüger, 2006; Lavialle-Defaix et al., 2006; Gautier et al., 2008). One of the most important key determinants of the DUM neuron excitability is the action potential threshold. The threshold determines when an action potential is initiated, sets the DUM neuron firing rate and shape neuronal computations including, for instance, temporal coding. Pacemaker activity in individual DUM neuron emerges from the concerted action of a complex complement of voltage-gated and background currents (Grolleau and Lapied, 2000; Wicher et al., 2001). However, voltage-gated currents activated near the action potential threshold are considered to be fundamental actors that contribute to controlling excitability. In cockroach isolated DUM neurons, different LVA channels are involved in the generation of the predepolarization, which regulate the firing frequency. In this preparation, two types of LVA calcium currents identified as transient and maintain calcium currents have specialized function in the spontaneous electrical activity. The LVA transient calcium current is involved in the first part of the predepolarization whereas the LVA maintained calcium current participates in the last two-thirds of the predepolarizing phase (Grolleau and Lapied, 1996, 2000; Wicher et al., 2001). Besides these two LVA calcium currents, a third unusual LVA inward current permeable to Na/Ca play an important role in pacemaking of DUM neurons (Defaix and Lapied, 2005). In fact, the activation of the LVA transient calcium current brings the membrane potential to the threshold of the LVA maintained Na/Ca current activation. This current leads to further depolarization, which allows to reach activation threshold of the LVA maintained calcium current. Together, these combined events produce the pre-depolarization (Figure 1C) essential for triggering DUM neuron pacemaker activity. Because the Na/Ca current is activated in an intermediate potential range between LVA transient and maintained calcium currents (i.e., subthreshold potential), it represents the LVA channel, which could be continuously and extensively modulated by a variety of intracellular signaling pathways including octopaminergic neuromodulator receptors, known to modulate spontaneous activity, as previously reported (Achenbach et al., 1997; Wicher et al., 2001). Although OA is known to modulate number of physiological and behavioral processes in invertebrates (Verlinden et al., 2010), there is, however, no data available to explain the modulatory action of OA in DUM neuron firing property.

It has been well established that there are significant similarities between the octopaminergic signaling pathways in invertebrates and the dopaminergic system in vertebrates (Roeder, 1999). The classification profile for OARs is based on the similarities of these receptors to vertebrate adrenergic receptors in terms of amino acid sequence and intracellular signaling pathways. Three classes of OARs have been characterized (Maqueira et al., 2005) and it has been reported, for instance, that activation of α-adrenergic-like OAR by OA results in an increase in intracellular levels of calcium and cAMP whereas β-adrenergic-like receptor activation only elevates cAMP concentrations (Bischof and Enan, 2004; Balfanz et al., 2005; Evans and Maqueira, 2005; Maqueira et al., 2005; Ohtani et al., 2006; Beggs et al., 2011). Based on these data, identifying the intracellular signaling pathway activated by vertebrate dopamine receptor stimulation could contribute to the understanding of the specific octopaminergic functions in insects. In this context, one of the most interesting features of insect-type octopaminergic receptors is that they could be indirectly coupled to the well-known vertebrate phosphoprotein DARPP-32 (Greengard et al., 1999). Because the phosphorylation of this protein is regulated by dopamine and cAMP, it is named DARPP-32 (Dopamine and cAMP-regulated phosphoprotein Mr 32,000). DARPP-32, expressed in different brain regions in vertebrates but also in peripheral organs such as kidney, adrenal medulla and parathyroid cells, plays a key role in mediating the biochemical, electrophysiological and behavioral of dopamine on dopaminoceptive neurons (Ouimet et al., 1984; Greengard et al., 1999; Svenningsson et al., 2004). Although DARPP-32 has also been implicated in mediating the actions of other neurotransmitters systems such as glutamate and serotonin, there is no information about the existence of such phosphoprotein in insects, mediating the action of OA via given OARs, which have a close pharmacological relationship with dopamine receptors.

In our study, we have demonstrated that OA modulates DUM neuron firing properties via the regulation of the LVA Na/Ca current through the participation of the phosphoprotein-like DARPP-32. Up to date, this is the first example reporting such physiological function for DARPP-32. Using specific pharmacological agents together with DARPP-32 and DARPP-32-P, we can propose the hypothetical scheme shown in Figure 6C. Like dopamine in vertebrates, OA acts on OARs using cAMP as a mediator in the process. Increased cAMP concentration, via AC, activates PKA, which induces DARPP-32 phosphorylation. The PKA-induced DARPP-32 phosphorylation converts this protein into a potent inhibitor of the protein phosphatase PP1/2A. The resulting inhibition of the phosphatase reduces Na/Ca current amplitude, which thereby decreases the DUM neuron pacemaker activity. DARPP-32 phosphorylation is opposed by a dephosphorylation process. For elevated intracellular calcium concentration, the dephosphorylation is catalyzed by a calcium/calmodulin-sensitive protein phosphatase PP2B. In this case, PP2B seems to play a prominent role in the regulation of the DARPP-32 phosphorylation and indirectly in the DUM neuron excitability. Based on the classification of the OARs linked to intracellular signaling pathways (i.e., cAMP and/or calcium; Evans and Maqueira, 2005; Maqueira et al., 2005), the results presented in this study help to understand better why OA, depending on the concentration tested (Achenbach et al., 1997; Wicher et al., 2001), increases or decreases the DUM neuron spontaneous electrical activity. Another interesting point raised is that DARPP-32, which is known to be a key cellular regulator, has been mainly characterized in mammals. Today, there is no comparative analysis of this phosphoprotein complex across other vertebrates and invertebrates. Understanding DARPP-32 function from the evolutionary perspective will help to further our understanding of the phylogenetic origins and evolutionary conservation of this protein. Based on our results, it appears that the phosphoprotein DARPP-32 could represent a more generic signaling motif for different living organisms including insects. On the other hand, the DARPP-32-dependent mechanism proposed here is quite generalizable to various systems. Thus, further exploration of the wider signaling network involved in this process is of interest. An important question, which is currently under investigation, is the unexpected regulation of the LVA maintained Na/Ca current by low internal ATP concentration. Additional experiments are in progress to clarify this point. Finally, it is known that several other crosstalk points exist between the calcium and OA signaling axis at various downstream levels of the synaptic signaling. Thus, phosphoprotein-dependent mechanisms could represent a more general central nervous system-wide signaling motif responsible for the implementation of network coding on sub-cellular signal integration of environmental cues.

## Author Contributions

BL designed the experiments, made analysis of the results and wrote the manuscript. AD conducted the experiments and made analysis of the results. MS and VR discussed the results and contributed to the text. EM contributed to the text.

## Conflict of Interest Statement

The authors declare that the research was conducted in the absence of any commercial or financial relationships that could be construed as a potential conflict of interest.
